# Epithelial NSD2 maintains FMO‐mediated taurine biosynthesis to prevent intestinal barrier disruption

**DOI:** 10.1002/ctm2.70128

**Published:** 2024-12-10

**Authors:** Yue Xu, Xiuying Xiao, Chunxiao Ma, Ziyi Wang, Wenxin Feng, Hanyu Rao, Wei Zhang, Ningyuan Liu, Rebiguli Aji, Xiangjun Meng, Wei‐Qiang Gao, Li Li

**Affiliations:** ^1^ State Key Laboratory of Systems Medicine for Cancer Renji‐Med X Clinical Stem Cell Research Center Ren Ji Hospital School of Medicine and School of Biomedical Engineering Shanghai Jiao Tong University Shanghai China; ^2^ School of Biomedical Engineering and Med‐X Research Institute Shanghai Jiao Tong University Shanghai China; ^3^ Department of Oncology Ren Ji Hospital School of Medicine Shanghai Jiao Tong University Shanghai China; ^4^ Gastroenterology Shanghai Ninth People's Hospital School of Medicine Shanghai Jiao Tong University Shanghai China; ^5^ Center for Digestive Diseases Research and Clinical Translation of Shanghai Jiao Tong University Shanghai China; ^6^ Shanghai Key Laboratory of Gut Microecology and Associated Major Diseases Research Shanghai China

**Keywords:** histone methylation, IBD, intestinal epithelial homeostasis, NSD2, taurine biosynthesis

## Abstract

**Background:**

Inflammatory bowel disease (IBD) presents a significant challenge due to its intricate pathogenesis. NSD2, a histone methyltransferase responsible for dimethylating histone 3 at lysine 36, is associated with transcriptional activation. NSD2 expression is decreased in both the intestinal epithelial cells (IECs) of IBD patients and the IBD mouse model. However, the precise role of NSD2 in IBD remains unexplored.

**Methods:**

Colon tissues from IBD mice, SW620 cells and MC38 cells, were used as research subjects. Clinical databases of IBD patients were analysed to investigate whether NSD2 expression is reduced in the occurrence of IBD. NSD2‐knockout mice were generated to further investigate the role of NSD2 in IBD. The IECs were isolated for RNA sequencing and chromatin immunoprecipitation sequencing to identify molecular signalling pathways and key molecules leading to IBD in mice. Molecular and cellular experiments were conducted to analyse and validate the role of NSD2 in the development of IBD. Finally, rescue experiments were performed to confirm the molecular mechanism of NSD2 in the development of IBD.

**Results:**

Deficiency of NSD2 in mouse IECs aggravated epithelial barrier disruption and inflammatory response in IBD. Mechanistically, NSD2 loss led to downregulation of H3K36me2 and flavin‐containing monooxygenase (FMO) (taurine‐synthesis enzyme) mRNA, resulting in decreased taurine biosynthesis in IECs. Significantly, supplementation with taurine markedly alleviated the symptoms of NSD2 deficiency‐induced IBD.

**Conclusions:**

These data demonstrate that NSD2 plays a pivotal role in maintaining FMO‐mediated taurine biosynthesis to prevent intestinal inflammation. Our findings also underscore the importance of NSD2‐H3K36me2‐mediated taurine biosynthesis in maintaining intestinal mucosal barrier homeostasis.

**Key points:**

- In this study, we investigated the role of the histone methyltransferase NSD2 in preventing intestinal barrier disruption by sustaining taurine biosynthesis. NSD2 levels were reduced in both human specimens and mouse models of IBD. We demonstrate that NSD2 loss hinders the process of taurine synthesis in intestinal cells, leading to increased intestinal inflammation.
- Supplementation with taurine significantly relieved the symptoms caused by NSD2 deficiency. These data suggest that maintenance of NSD2‐mediated taurine biosynthesis is vital for preserving the intestinal barrier and attenuating inflammation.

## Graphical abstract

- NSD2 deficiency in mouse IECs aggravated epithelial barrier disruption and inflammatory response in IBD.
- NSD2 loss downregulated H3K36me2 and FMO (taurine‐synthesis enzyme) mRNA, resulting in decreased taurine biosynthesis in IECs.
- Supplementation with taurine significantly attenuated the symptoms of IBD caused by NSD2 deficiency.

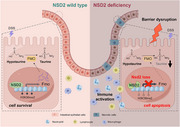

## 1 INTRODUCTION

Inflammatory bowel disease (IBD), which includes Crohn's disease (CD) and ulcerative colitis (UC), represents a group of chronic inflammatory diseases affecting the gastrointestinal tract, distinguished by persistent immune activation, mucosal inflammation and tissue damage.^1^ The intact epithelium and mucosal barrier collaborate to maintain the integrity of the intestinal barrier and colonic homeostasis. The breakdown of the epithelial barrier leads to the inappropriate movement of gut luminal contents, commensal microbiota and pathogenic microbes into the intestinal lamina propria. Epithelial barrier dysfunctions causes intestinal inflammation and mucosal injury, ultimately causing intestinal carcinogenesis that affects millions of people worldwide.^2^ Despite the recognised importance of the epithelial barrier, the complex interplay of molecular and cellular pathways in IBD pathogenesis remains poorly understood.

Epigenetic regulation, encompassing DNA methylation, histone modification, non‐coding RNA and chromatin interaction, is critical for controlling gene expression changes in gastrointestinal development and homeostasis.^3^, ^4^ Aberrant epigenetic modifications have been implicated in the pathogenesis of gastrointestinal diseases, such as IBD and colorectal cancer (CRC), where they contribute to disease progression.^5^, ^6^, ^7^, ^8^, ^9^, ^10^ Notably, recent studies have highlighted the importance of histone modifications in the occurrence and development of IBD,^4^ suggesting that targeting these epigenetic marks might be a promising therapeutic strategy for the treatment of gastrointestinal diseases.

NSD2, also known as Wolf‐Hirschhorn syndrome candidate gene‐1 (WHSC1) or MM overexpresses multiple myeloma SET domain‐containing protein (MMSET), encodes a histone methyltransferase that specifically dimethylates histone H3 at lysine 36 (H3K36me2).^11^ NSD2 plays a pivotal role in regulating gene expression by modulating chromatin accessibility and altering nucleosome structure.^12^, ^13^, ^14^ It is frequently found to be aberrantly expressed, amplified or somatically mutated in various types of cancer. Notably, in CRC, dysregulation of NSD2 promotes colon rectal tumour angiogenesis and is associated with a poor prognosis.^15^ Furthermore, NSD2 is crucial for maintaining the intestinal barrier in metabolic diseases.^16^ However, the role of NSD2 in intestinal inflammation, particularly its impact on IBD, remains understudied.

Amino acid metabolism, particularly taurine, has been shown to be integral to preserving intestinal epithelial homeostasis.^17^ As one of the most abundant amino acids, taurine can remodel the intestinal microbiota, enhancing infection resistance and bolstering antitumour immunity.^18^, ^19^ The potential impact of taurine on IBD is an area of growing interest, yet the precise mechanisms by which taurine metabolism influences IBD pathology are not fully understood.

In this work, we aimed to investigate the role of NSD2 in IBD by analysing the expressions of NSD2 in IBD patients and mouse models. Using a conditional Nsd2^Vil‐KO^ mouse line and IEC‐derived organoids, we demonstrate that NSD2 is reduced in epithelial cells of patients with IBD. We revealed that NSD2‐mediated taurine biosynthesis is essential for the formation of proper intestinal barrier function. Furthermore, we show that deficiency of NSD2 in mouse IECs aggravates epithelial barrier disruption and inflammatory response in IBD. Our findings emphasise that NSD2 plays a crucial role in regulating IEC apoptosis and intestinal inflammation, offering new perspectives on potential therapeutic approaches for IBD treatment.

## 2 MATERIALS AND METHODS

### 2.1 Mice

All mice were maintained in a specific‐pathogen‐free facility and all mouse experimental protocols were approved by the Renji Hospital Animal Care and Use Committee (202201027). NSD2‐flox mice (Nsd2^f/f^) and NSD2‐overexpressing mice (Nsd2^OE/+^) were generated as previously reported^20^ and generously provided by Prof. Jun Qin. Villin‐Cre mice were procured from Shanghai Model Organisms Co. Ltd. By crossing these lines, we produced the experimental groups, encompassing the genotypes: Nsd2^Vil‐KO^ and Nsd2^Vil‐OE^. The mice were sacrificed at the indicated time for the examination of intestinal histology. Animals experiencing greater than a 20% reduction in body weight over a 7‐day period were humanely euthanised and categorised as deceased. All the mice were bred on a C57BL/6J background and littermates with the same treatment were used for control experiments. In the context of permeability assays, mice at 2 months of age were subjected to a 4‐h fast prior to being administered with Fluorescein Isothiocyanate (FITC)‐labelled dextran (at a dosage of 500 mg/kg of body weight). The measurement of fluorescence intensity was based on a standard curve specific to FITC‐dextran. To induce colitis, mice at 2 months of age were fed 2% dextran sodium sulphate (DSS)^21^ (molecular weight, 36–50 kDa; MP Biomedicals) for 5 days, followed by regular drinking water. Body weight was recorded daily. To investigate the protective capability of taurine, the mice were exposed to 2% DSS for 5 days, and the taurine supplementation group was given 1000 mg/kg taurine by intragastric administration daily. The control group was given distilled water (*n* = 3 for each group). At the end of the experimental period, the animals were euthanised, and the intestine were collected for the following experiments.

### 2.2 Human specimen analysis

Patients with IBD and non‐IBD control subjects for this study were recruited from Renji Hospital. The use of pathological specimens, as well as the review of all pertinent patient records, were approved by the Ethics Review Board of Renji Hospital. Immunohistochemical analyses were performed using anti‐Nsd2, Abcam, Cat# ab75359 (1:500); anti‐H3K36me2, Abcam, Cat# ab9049 (1:1000). Protein expression was assessed using a 10‐point staining index, calculated by multiplying the average staining intensity (0–3) by the extent of staining (0–3), resulting in a range from 0 to 9. Expression levels from 0 to 4 were considered low, while levels from 5 to 9 were classified as high.

### 2.3 Cell culture, plasmids and transfections

Human colorectal adenocarcinoma cell line SW620 and mouse colorectal cell line MC38 were cultured in Dulbecco's Modified Eagle Medium (DMEM) supplemented with 1% penicillin‒streptomycin and 10% foetal bovine serum (FBS) at 37°C in an atmosphere of 5% CO_2_. Stable transfection was achieved by exposing cells to viral vectors and selecting with 2 µg/mL puromycin for a period of 7 days. The human FMO2 and FMO4 pCMV‐Entry vectors were procured from Hunan Fenghui Biotechnology Co., Ltd. The transfection of cells with these plasmids was facilitated by the use of the EZ transfection regimen.

### 2.4 Taurine measurements

Levels of taurine were detected in the IECs and cell lines by Taurine Microplate Assay Kit (Absin) according to the manufacturer's instructions.

### 2.5 IEC isolation

IECs were isolated from Nsd2^Vil‐KO^, Nsd2^f/f^, Nsd2^Vil‐OE^ and Nsd2^OE/+^ mice. IECs were extracted from the colonic tissue of mice. The harvested colonic tissue from the mice was dissected into minute pieces measuring approximately 1 mm and then incubated at a temperature of 37°C for a quarter of an hour with a solution containing 8 mM ethylenediaminetetraacetic acid (EDTA). Following this incubation period, the EDTA solution was decanted and replaced with phosphate buffered saline (PBS), after which the mixture was agitated with force for a duration of 45 s. This process was reiterated, and the supernatants collected on both occasions were pooled together. After the filtration step through a 100‐µm cell strainer, the combined supernatant was subjected to centrifugation at a rotational speed of 2000 rpm at a chilly temperature of 4°C for 2 min. The supernatant was then carefully decanted, leaving behind the sedimented cells, which were subsequently re‐suspended in chilled PBS.

### 2.6 Organoid culture and analysis

The intestinal tracts were longitudinally dissected, and the protruding villi were removed. Once thoroughly rinsed in chilled PBS, the segments were immersed in a solution of 2 mM EDTA/PBS for a decade at a temperature of 4°C. Subsequently, the EDTA solution was siphoned off and substituted with PBS, followed by vigorous agitation for a duration of 45 s. The crypt isolates were then refined through a series of centrifugation procedures. One hundred microlitres of advanced DMEM/F12 medium (provided by Invitrogen) enriched with growth factors—comprising 50 ng/mL of EGF (PeproTech), 500 ng/mL of R‐spondin (PeproTech) and 100 ng/mL of Noggin (PeproTech)—was introduced, with the medium being renewed every 2‒3 days. Following a 48‐h period, the organoids underwent staining with 7‐AAD for a brief interval of 5 min, after which images were captured and the data were quantified utilising ImageJ software.

### 2.7 RNA sequencing and data analysis

Total RNA was purified using Trizol reagent (Life Technologies Corp.), followed by DNase treatment to eliminate any genomic DNA contamination. Messenger RNA was isolated via the NEBNext PolyA mRNA Magnetic Isolation Module (New England Biolabs), and subsequently utilised for the construction of RNA sequencing (RNA‐seq) libraries with the NEB Next Ultra Directional RNA Library Prep Kit for Illumina (New England Biolabs). The libraries were sequenced on an Illumina platform using a paired‐end 2 × 150 bp protocol. Raw sequencing reads were refined to yield high‐fidelity clean reads by eliminating sequencing adapters, reads shorter than 35 nucleotides, and reads of poor quality, employing Cutadapt (version 1.9.1) and Trimmomatic (version 0.35). Subsequently, FastQC was applied to confirm the quality of the reads. The clean reads were aligned to the mouse reference genome (GRCm38 assembly) using HISAT2 software. The quantification of gene expression was conducted using the FPKM method (fragments per kilobase of exon per million mapped fragments) with StringTie.^22^ Differential gene expression analysis was performed using the Ballgown R package.^23^ To account for multiple comparisons, the false discovery rate method was employed to determine the adjusted *p*‐values, assessing the statistical significance of observed differences. Here, genes with an adjusted *p*‐value of less than  .05 were selected for further analysis.

### 2.8 Chromatin immunoprecipitation sequencing data analysis and chromatin immunoprecipitation‐qPCR validation

Cells were fixed with a 1% formaldehyde solution for 10 min at ambient temperature and then treated with 125 mM glycine to terminate the crosslinking reaction. The sonicated chromatin fragments were cleared of debris and subjected to immunoprecipitation using Protein A + G Magnetic beads conjugated to anti‐H3K36me2 antibodies (Abcam, Cat# ab9049). Post‐reverse crosslinking, both chromatin immunoprecipitation (ChIP) and input DNA fragments underwent end‐repair and adenine tailing using the NEBNext End Repair/dA‐Tailing Module (E7442, NEB), followed by adapter ligation with the NEBNext Ultra Ligation Module (E7445, NEB). The DNA libraries were amplified for 15 cycles and sequenced on an Illumina NextSeq 500 platform using a single‐end 1 × 75 bp protocol. Raw sequencing reads were processed to extract high‐quality clean reads by removing sequencing adapters, short reads (length < 35 bp), and low‐quality reads with Cutadapt (version 1.9.1) and Trimmomatic (version 0.35). FastQC was utilised to verify the quality of the reads. The clean reads were aligned to the mouse reference genome (GRCm38 assembly) using Bowtie2 software (version 2.2.6). Peak detection was carried out using the MACS algorithm (version 2.1.1) with a *p*‐value threshold set at  .01. The annotation of peak sites relative to gene features was accomplished using the ChIPseeker R package.^24^ The ChIP‐qPCR assays were executed utilising the EZ ChIP kit (Millipore). The methodology adhered to the instructions provided by the kit manufacturer. In summary, the isolated IECs were crosslinked using a 1% formaldehyde solution and then sheared through sonication. For the immunoprecipitation step, an antibody against H3K36me2 (Abcam, Cat# ab9049) was employed. Once the samples were washed and underwent reverse crosslinking, the enriched DNA was subjected to amplification with specific primers and subsequently quantified via qPCR. The sequences of the primers are detailed in Table S1.

### 2.9 Histology, haematoxylin–eosin staining and immunohistochemistry

Tissues were preserved in a solution of 4% paraformaldehyde for an entire night, employing the Swiss roll method, subsequently encapsulated in paraffin, and sectioned into slices measuring 7 µm in thickness. Sections treated with haematoxylin and eosin staining were appraised by a pathologist in a masked evaluation. The colitis scores were determined by a composite index that encompassed the intensity of inflammation, ulceration and mucosal hyperplasia, each graded on a scale from 0 to 4.^25^ For the enumeration of goblet cells, periodic acid‐Schiff (PAS)‐positive cells were tallied across five arbitrary fields from a minimum of five mice per genetic configuration. The quantification of Paneth cells involved counting lysozyme‐positive cells within 100 crypts from no fewer than three mice per genetic makeup. The presented data are depicted as the mean ± SEM. The assessment of statistical significance across different groups was conducted using the chi‐squared test. The primary antibodies utilised for immunohistochemistry (IHC) included: anti‐Ki67 (B56), BD Biosciences, Cat# 550609 (1:500); anti‐Nsd2, Abcam, Cat# ab75359 (1:500); anti‐H3K36me2, Abcam, Cat# ab9049 (1:1000). The biotinylated secondary antibodies were acquired from Jackson Immunology. The staining was rendered visible with ABC Kit Vectastain Elite (Vector Laboratories) and DAB substrate (Vector Laboratories). The PAS staining was implemented using kits from Sigma‒Aldrich, and the alkaline phosphatase activity assay was executed following the protocols provided by the manufacturer, with the Alkaline Phosphatase Staining Kit from Vector Laboratories.

### 2.10 Immunofluorescence

The sections underwent the process of deparaffinisation and rehydration prior to the retrieval of antigens, following which they were allowed to reach ambient temperature. Subsequently, the permeabilisation of the cell membranes was carried out with a  .5% solution of Triton X‐100, which was succeeded by a rinse in PBS and a blocking step involving 10% Bovine Serum Albumin (BSA). Thereafter, the primary antibody was applied to the tissue samples and left to incubate at a temperature of 4°C throughout the night. The samples were then subjected to nuclear counterstaining with a DNA‐specific fluorescent probe (DAPI), a DNA‐specific fluorescent probe, after their exposure to the secondary antibody. Primary antibodies used for IF were as follows: anti‐Ki67 (B56), BD Biosciences, Cat# 550609 (1:500); anti‐ZO‐1, Invitrogen, Cat# 40‐2200 (1:100); anti‐E‐cadherin (24E10), Cell Signaling Technology, Cat# 3195T (1:1000); anti‐cleaved caspase‐3 (Asp175), Cell Signaling Technology, Cat# 9661 (1:1000); anti‐lysosome, DAKO, Cat# A0099 (1:500); anti‐ChgA, Abcam, Cat# ab715 (1:100); anti‐Mucin2 (Ccp58), Santa Cruz Biotechnology, Cat# sc‐7314 (1:500); and anti‐Lgr5, Abcam, Cat# ab75732 (1:100).

### 2.11 Immunoblotting

The primary antibodies used in this study were as follows: anti‐NSD2 (29D1), Abcam, Cat# ab75359 (1:500); anti‐H3K36me2, Abcam, Cat# ab9049 (1:1000); anti‐H3, Abcam, Cat# ab10799 (1:1000); anti‐caspase‐3 (8G10), Cell Signaling Technology, Cat# 9665 (1:1000); anti‐C‐caspase‐3, Cell Signaling Technology, Cat# 9661 (1:1000); anti‐PARP (46D11), Cell Signaling Technology, Cat# 9532 (1:1000); anti‐ZO1, Invitrogen, Cat# 33‐9100 (1:500); anti‐PCNA, Santa Cruz Biotechnology, Cat# SC‐7909 (1:1000); and anti‐E‐cadherin, Cell Signaling Technology, Cat# 3195T (1:1000). Raw data of immunoblotting can be found in the source data file. Representative data are shown from one out of three independent experiments with similar results.

### 2.12 FACS

Cell suspensions underwent analysis through flow cytometry. In terms of the gating strategy, initially, the forward scatter (FSC)/side scatter (SSC) criteria were utilised to delineate the viable cell population. Subsequently, the gating was refined based on the specific fluorochrome for further examination. Consistent parameters were employed for gating across all samples within the same set of experiments and comparative analyses. The antibodies used in this study were as follows: anti‐CD11b‐APC, eBioscience, Cat# 47‐0112‐82 (1:400); anti‐F4/80‐PE, eBioscience, Cat# 25‐4801‐82 (1:400); anti‐CD4‐FITC, eBioscience, Cat# 11‐5040‐41 (1:400); and Gr‐1‐FITC, eBioscience, Cat# 11‐0114‐82 (1:400).

### 2.13 Isolation of lamina propria cells and analysis

The colonic tissue was dissected into minute fragments and subsequently immersed in Roswell Park Memorial Institute (RPMI) 1640 medium enriched with FBS,  .5 mM dithiothreitol, 5 mM EDTA‒PBS and antimicrobial agents at a temperature of 37°C for a duration of 30 min. Following the excision of the epithelial stratum, the residual colonic segments were further incubated at 37°C with RPMI 1640 medium that included Collagenase IV (Roche) and DNase (Roche) for an additional 30 min. The cells isolated from the lamina propria were then labelled with antibodies against surface markers, specifically CD4‐FITC for CD4, CD11b‐APC for CD11b, F4/80‐PE for F4/80 and Gr‐1‐FITC for Gr‐1.

### 2.14 Statistical analysis

All experimental procedures were conducted with a cohort of 3–15 mice or, at a minimum, were replicated in three separate experimental runs. Data are generally depicted as the mean ± SEM, and the determination of statistical significance was made employing a two‐tailed Student's *t*‐test. Correlation analyses were performed using Pearson's correlation coefficients to assess the association between NSD2 expression and gene expression levels. The chi‐squared test was utilised to ascertain whether there existed a substantial divergence between anticipated and actual frequencies within one or more classification categories. The significance levels are denoted as follows: ^*^ *p* < .05, ^**^ *p* < .01 and ^***^ *p* < .001. For a detailed reporting summary, refer to the Nature Research Reporting Summary associated with this manuscript for further insights into the research design.

## 3 RESULTS

### 3.1 NSD2 expression is decreased in IBD

**FIGURE 1:**
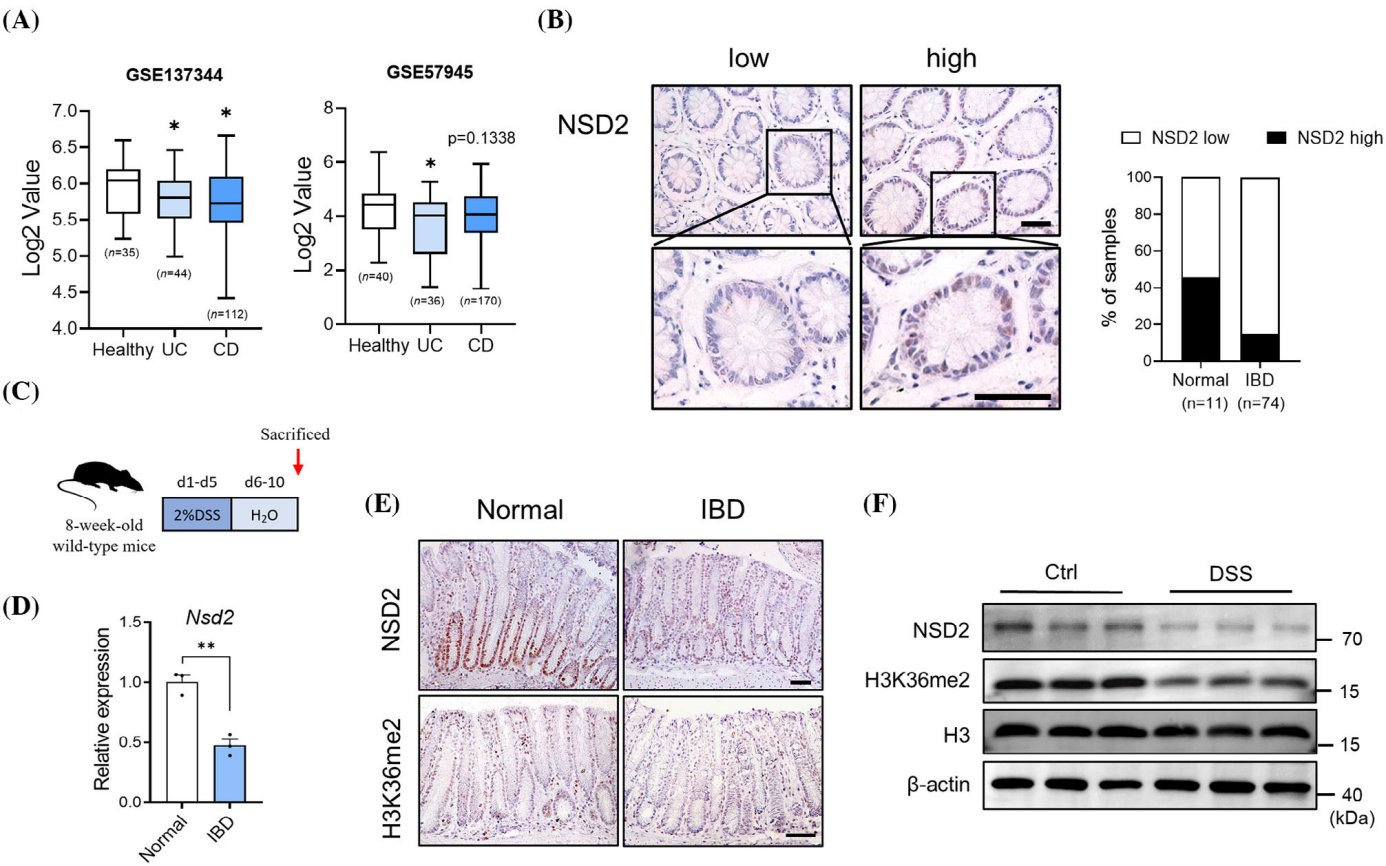
NSD2 expression is decreased in inflammatory bowel disease (IBD). (A) Box plot of NSD2 mRNA in healthy controls, ulcerative colitis (UC) and Crohn's disease (CD) specimens (using datasets GSE137344 and GSE57945). (B) NSD2 staining images are shown in the left panel, and epithelial NSD2 expressions in normal and IBD biopsies are quantified in the right panel (chi‐squared test). Staining indexes use a 10‐point quantification scale, and a score >4 is considered a higher level. (C) Schematic representation of the dextran sodium sulphate (DSS) protocol used to induce acute colitis. (D‒F) samples are all derived from 8‐week‐old wild‐type mice with or without DSS treatment. (D) RT‐qPCR analysis of NSD2 mRNA in intestinal epithelial cells (IECs) from control and DSS‐treated wild‐type mice (*n* = 5 per group). Immunohistochemical (E) and immunoblot analyses (F) of NSD2 expression in control and DSS‐treated wild‐type mice are shown. Scale bar: 50 µm. The data represent the mean ± SEM, and statistical significance was determined by a two‐tailed Student's *t*‐test unless otherwise indicated. ^*^ *p* < .05, ^**^ *p* < .01 and ^***^ *p* < .001; N.S., not significant.

To investigate the role of NSD2 in the pathogenesis of IBD, we examined the expression of NSD2 mRNA in publicly available gene datasets of CD and UC samples. The results indicated that NSD2 mRNA was downregulated in CD and UC specimens compared to those in healthy controls (Figure 1A). To assess the clinical significance of NSD2 in IBD, we conducted IHC analyses to evaluate the protein level of NSD2. As shown in Figure 1B, the expression of NSD2 in IBD patients biopsy specimens was significantly lower than normal colonic epitheliam. Similar to the observations in human IBD, the expression levels of NSD2 and H3K36me2 were also decreased in DSS‐induced IBD mice at both the mRNA and protein levels compared to controls (Figure 1C‒F). Taken together, these results suggested a potential link between the NSD2 expression and IBD pathogenesis.

### 3.2 NSD2 deficiency in IECs exacerbates intestinal inflammatory responses

**FIGURE 2:**
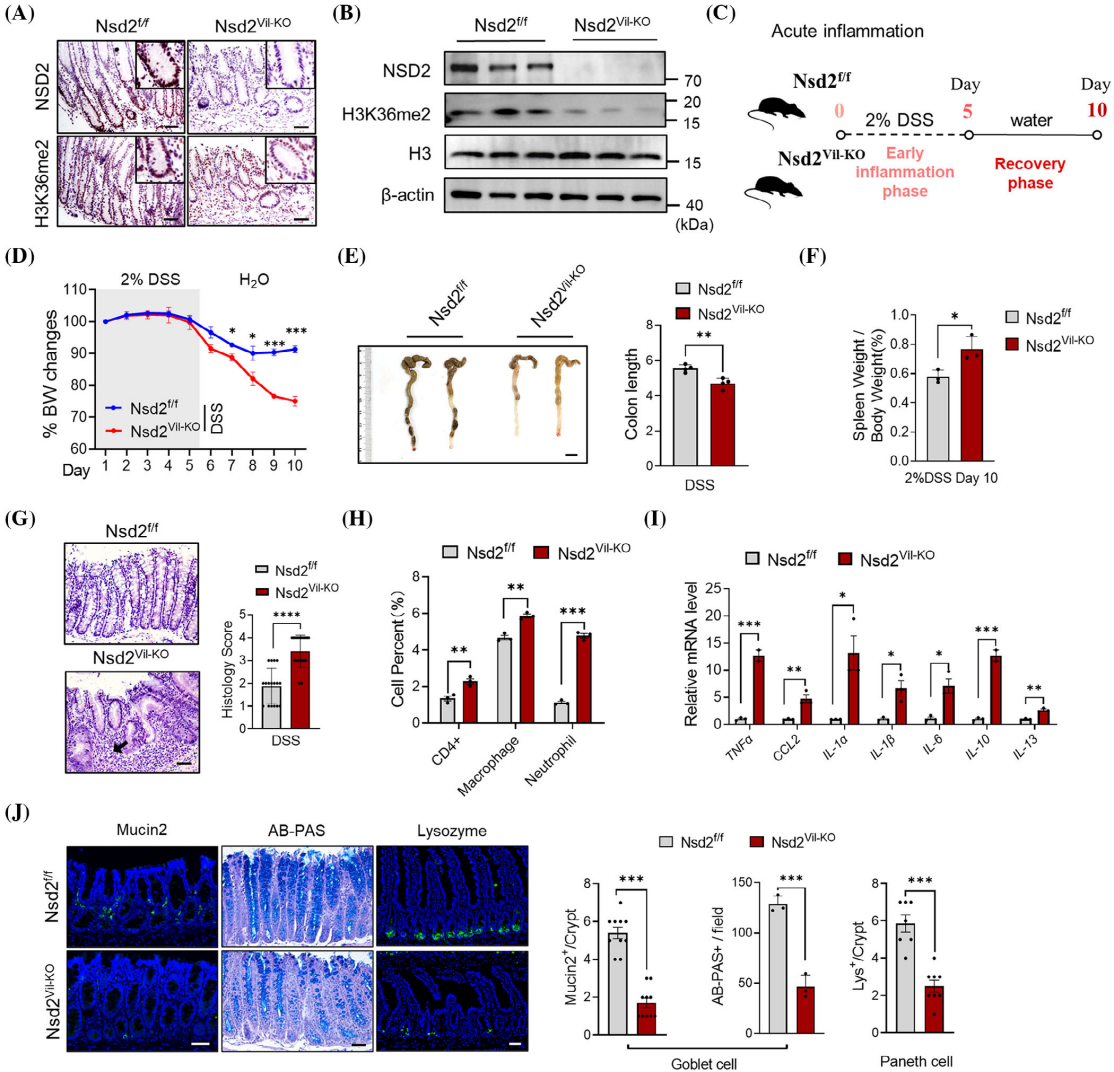
NSD2 deficiency in intestinal epithelial cells (IECs) exacerbates intestinal inflammatory responses. (A and B) Immunohistochemical (A) and immunoblot (B) analyses of NSD2 and H3K36me2 expression in the IECs from Nsd2^f/f^ and Nsd2^Vil‐KO^ mice (*n* = 5 per group). (C) Schematic representation of the dextran sodium sulphate (DSS) protocol used to induce acute colitis. (D) Mice were fed with 2% DSS solution to induce colitis, and weight loss was recorded (*n* = 4). Scale bar: 1 cm. (E) Representative images of colons from *Nsd2* ^f/f^ and Nsd2^Vil‐KO^ mice after DSS treatment. (F) Spleen weight normalised to body weight at the time the mice were killed (day 10). (G) Haematoxylin and eosin (H&E)‐stained sections and the quantitation of histology score of colon tissue collected on day 10 from 2% DSS‐treated Nsd2^f/f^ and Nsd2^Vil‐KO^ mice are shown. Scale bar: 50 µm. (H) After DSS treatment, colonic lamina propria cells from Nsd2^f/f^ and Nsd2^Vil‐KO^ mice are analysed by flow cytometry (*n* = 3). (I) Relative mRNA expression levels of inflammatory cytokines and chemokines in the whole colon of Nsd2^f/f^ and Nsd2^Vil‐KO^ mice were determined by RT‐qPCR (*n* = 3). (J) Mucin2 (goblet cell), Alcian blue‐periodic acid‐Schiff (AB‐PAS; goblet cell) and lysozyme (Lys; Paneth cell) staining of the intestines from 2% DSS‐treated mice and quantitation results are shown on the right (*n* = 3). Scale bar: 50 µm. All data are presented as mean ± standard deviation (SD), and statistical significance was determined by a two‐tailed Student's *t*‐test unless otherwise indicated. ^*^ *p* < .05, ^**^ *p* < .01 and ^***^ *p* < .001; N.S., not significant.

The above results prompted us to use genetically modified mouse models to investigate the potential function of NSD2 in colon inflammation. Nsd2^flox/flox^ (Nsd2^f/f^) mice and Villin‐Cre mice were crossed to generate Nsd2^flox/flox^ and Villin‐Cre (Nsd2^Vil‐KO^) mice. Western blotting and IHC staining results showed that NSD2 was efficiently deleted and the protein level of H3K36me2 was dramatically downregulated in Nsd2^Vil‐KO^ mice (Figure 2A,B). Meanwhile, the expression levels of NSD1 and NSD3 remained unchanged (Figure S1A). NSD2‐knockout mice do not exhibit spontaneous inflammation (Figure S1B,C), and there were no discernible differences in the number of stem/progenitor cells and terminally differentiated cells between wild‐type and Nsd2^Vil‐KO^ mice under steady‐state conditions (Figure S1D,E). To further investigate the role of NSD2 in IBD, we administered 2% DSS to induce IBD as widely reported in the drinking water of wild‐type and Nsd2^Vil‐KO^ mice as described^21^, ^26^ (Figure 2C). Nsd2^Vil‐KO^ mice exhibited significantly exacerbated IBD compared to control mice, as indicated by accelerated and more pronounced weight loss (Figure 2D), as well as enhanced colon shortening and spleen swelling on day 10 when they were sacrificed (Figure 2E,F). Histopathological examinations revealed that Nsd2^Vil‐KO^ mice exhibited more severe phenotypic changes, as evidenced by inflammatory infiltrates, crypt erosion and an increase in histology score (Figure 2G).

We then analysed immune cell infiltration using flow cytometry. After 5 days of DSS treatment, the numbers of CD4^+^ T cells, macrophages and neutrophils were increased in Nsd2^Vil‐KO^ mice (Figure 2H). As expected, DSS‐treated Nsd2^Vil‐KO^ mice exhibited an excessive immune response. RT‐qPCR quantitation confirmed a significant increase in the local production of pro‐inflammatory cytokines (*Il1a*, *Il1b*, *Il6*, *Il10*, *Il13*, *Tnfa* and *Ccl2*) in the colon of Nsd2^Vil‐KO^ mice (Figure 2I).

Additionally, NSD2 deletion results in a loss of IECs after DSS treatment (Figure S2). The loss of NSD2 significantly decreased the expression of Mucin2 (Muc2) and Alcian blue‐periodic acid‐Schiff (AB‐PAS), which are produced by goblet cells and form the structure of the intestinal mucus (Figure 2J). Lysozyme staining also showed that Nsd2^Vil‐KO^ mice had a reduced number of Paneth cells producing antimicrobial peptides in the small intestine compared to the wild‐type controls (Figure 2J). Collectively, our results demonstrated that epithelial NSD2 serves as a defense mechanism to prevent DSS‐induced IBD.

### 3.3 Epithelial NSD2 deletion exacerbates barrier dysfunction and cell apoptosis

**FIGURE 3:**
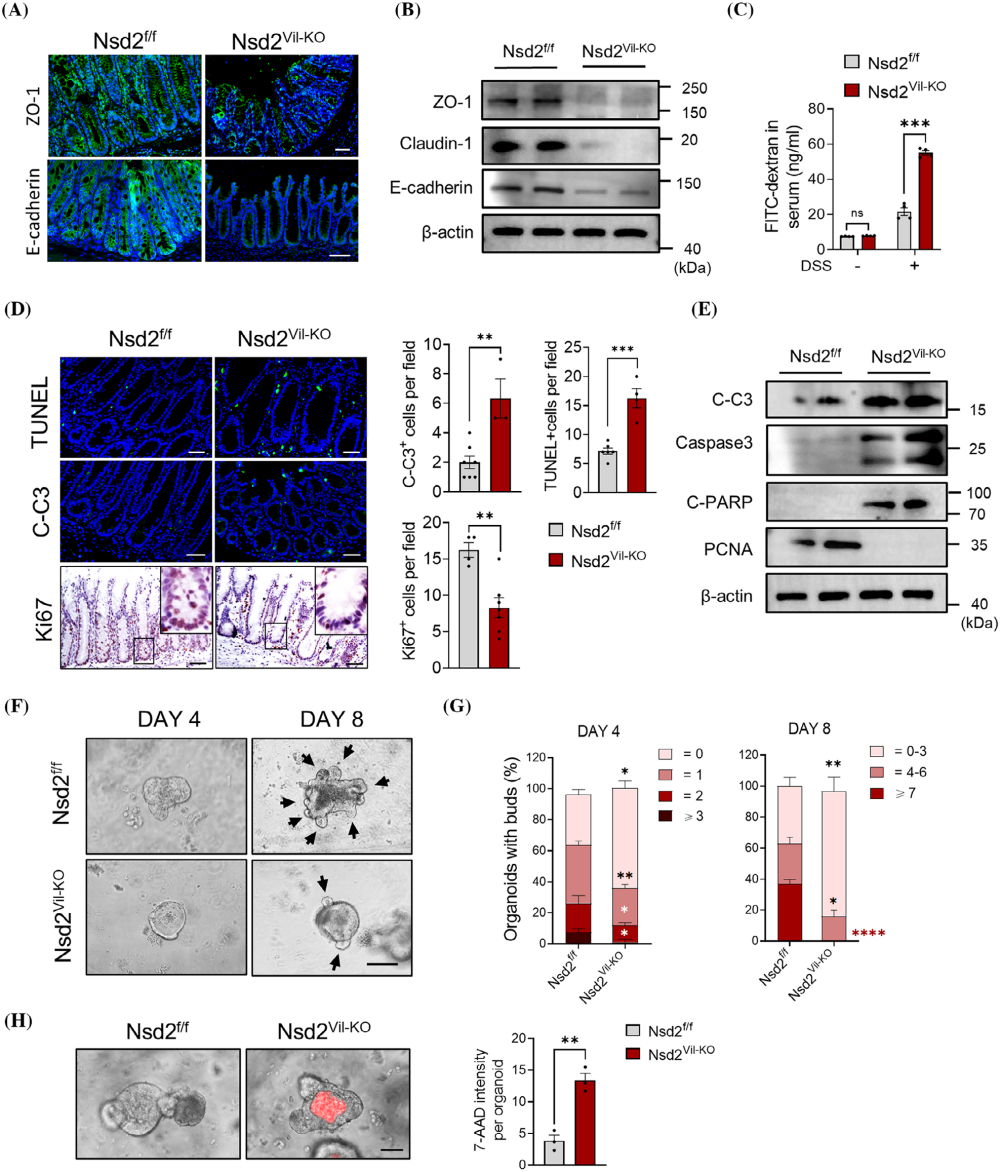
Epithelial NSD2 deletion exacerbated barrier dysfunction and cell apoptosis. (A and B) Representative immunofluorescence staining (A) and immunoblots (B) of ZO‐1 and E‐cadherin in colon sections from dextran sodium sulphate (DSS)‐treated Nsd2^f/f^ and Nsd2^Vil‐KO^ mice (*n* = 5 per group). Scale bar: 50 µm. (C) Colonic permeability was measured by the concentration of FITC‐dextran in the blood serum (*n* = 4). (D) TUNEL (upper), C‐C3 (medium) and Ki67 (lower) staining of colon sections and quantitation results are shown (*n* = 3). Scale bar: 50 µm. (E) Western blotting analysis of intestinal epithelial cells (IECs) isolated from Nsd2^f/f^ and Nsd2^Vil‐KO^ mice. (F) Representative images of intestinal organoids derived from the Nsd2^f/f^ and Nsd2^Vil‐KO^ mice on days 4 and 8. (G) Structure quantification of organoids on days 4 and 8 (*n* = 5, 30 organoids per mouse). Bars: 100 µm. Quantification was done by measuring the number of the buds. (H) Apoptotic cells (7‐AAD‐stained red) in organoids were imaged after stained with 7‐AAD for 24 h on day 6. Quantitation of the fluorescence density per organoid is shown on the right. The data represent the mean ± SEM, and statistical significance was determined by a two‐tailed Student's *t*‐test unless otherwise indicated. ^*^ *p* < .05, ^**^ *p* < .01 and ^***^ *p* < .001; FITC, Fluorescein Isothiocyanate; N.S., not significant.

Tight junctions are the primary determinants of barrier function in intact epithelia. Intestinal epithelial damage, such as erosions and ulcers, leads to the loss of tight junctions and therefore causing defects in local barrier function.^27^ To investigate the impact of NSD2 on intestinal barrier function in DSS‐induced IBD, we examined the expression level and localisation of the tight junction protein 1 (ZO‐1) and epithelial cadherin protein (E‐cadherin) in Nsd2^f/f^ and Nsd2^Vil‐KO^ mice. As shown in Figure 3A, Nsd2^Vil‐KO^ mice exhibited partially disrupted or discontinuous ZO‐1 staining, as well as a substantial reduction in E‐cadherin. In addition, Western blot analyses confirmed the reduction of ZO‐1, E‐cadherin and Claudin‐1 proteins in Nsd2^Vil‐KO^ mice (Figure 3B). Furthermore, our results suggested that NSD2 ablation resulted in colonic leakage, as evidenced by the higher serum FITC‐dextran concentrations detected in Nsd2^Vil‐KO^ mice compared to the controls after DSS treatment (Figure 3C). These findings demonstrated that NSD2 is necessary for maintaining mucosal integrity in an inflammatory environment.

Intestinal epithelial damage, accompanied by IEC shedding and oligocellular wounds, prompted us to investigate potential defects in cell survival in Nsd2^Vil‐KO^ mice.^27^ Compared to Nsd2^f/f^ mice, Nsd2^Vil‐KO^ mice showed significantly higher numbers of TUNEL‐ and cleaved caspase‐3‐positive cells, indicating heightened apoptosis (Figure 3D). This observation was corroborated by Western blot analysis, which demonstrated the activation of the pro‐apoptotic pathway, specifically caspase‐3, in colonic crypts of Nsd2^Vil‐KO^ mice (Figure 3E). Meanwhile, a reduction in Ki67‐positive cells was detected in the Nsd2^Vil‐KO^ mice (Figure 3D,E), suggesting that NSD2 deficiency not only promotes intestinal epithelial cell (IEC) death but also impairs epithelial cell self‐renewal.

We established intestinal organoid cultures to further validate these results. Consistently, loss of NSD2 led to a decrease in size and complexity (as evidenced by a lower number of buds; Figure 3F,G) and an increase in cell apoptosis of intestinal organoids (Figure 3H). These results collectively suggest that NSD2 is a critical regulator of IEC survival and proliferation.

### 3.4 NSD2 overexpression in IECs prevents DSS‐induced IBD

**FIGURE 4:**
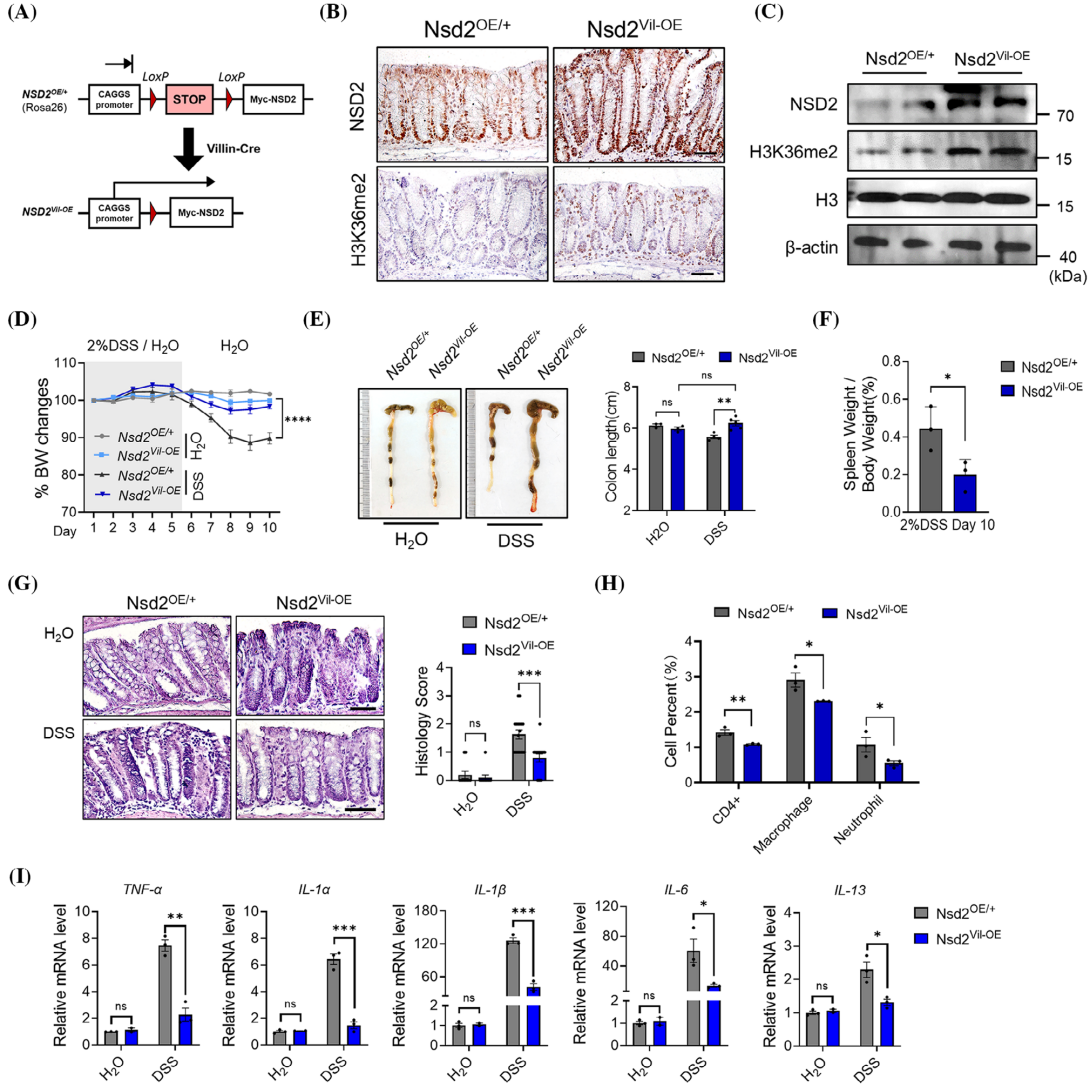
NSD2 overexpression in intestinal epithelial cells (IECs) prevented dextran sodium sulphate (DSS)‐induced inflammatory bowel disease (IBD). (A) Scheme of Nsd2^OE/+^ mice and conditional overexpression of NSD2 in IECs (Nsd2^Vil‐OE^) mice (*n* = 5 per group). (B) Immunofluorescent staining of NSD2 in colon sections from Nsd2^OE/+^ and Nsd2^Vil‐OE^ mice. Scale bar: 50 µm. (C) Immunoblots for NSD2 and H3K36me2 in colonic epithelial cells isolated from colitis mice. (D) Body weight change of mice treated with or without 2% DSS (*n* = 5). (E) The colon length of Nsd2^OE/+^ and Nsd2^Vil‐OE^ mice on day 10. (F) Spleen weight normalised to body weight at the time the mice were killed. (G) Representative haematoxylin and eosin (H&E) staining images of Nsd2^OE/+^ and Nsd2^Vil‐OE^ mice with or without DSS treatment. The histology score is shown on the right. Scale bar: 50 µm. (H) Colonic lamina propria cells from DSS‐treated Nsd2^OE/+^ and Nsd2^Vil‐OE^ mice are analysed by flow cytometry (*n* = 3). (I) Relative mRNA levels of proinflammatory cytokines in the colon tissue measured by RT‐qPCR (*n* = 3). Data are represented as mean ± standard deviation (SD). N.S., not significant; ^*^ *p* < .05, ^**^ *p* < .01 and ^***^ *p* < .001.

To investigate whether the upregulation of NSD2 could protect the mice from DSS‐induced IBD, we generated the mice that overexpress NSD2 specifically in IECs. Mice harbouring a single copy of a minigene consisting of a CAGGS promoter (a hybrid of chicken β‐actin and cytomegalovirus), a loxP‐STOP‐loxP (LSL) cassette and Myc‐tagged Nsd2 cDNA knocked into the Rosa26 locus (Nsd2^OE/+^)^20^ were crossed with Villin‐Cre mice to obtain Villin‐Cre; Nsd2^OE/+^ mice (referred to as Nsd2^Vil‐OE^ mice) (Figure 4A). The overexpression of NSD2 in the Nsd2^Vil‐OE^ mice was confirmed using IHC and Western blotting (Figure 4B,C).

Nsd2^Vil‐OE^ mice and littermates were treated with DSS as described above. By day 10, wild‐type mice exhibited IBD syndrome, as indicated by diarrhoea, significant body weight loss, and pronounced shortening and thickening of the colon. In contrast, Nsd2^Vil‐OE^ mice developed virtually no symptoms, and their large bowel showed few signs of IBD, even compared to the control group that did not receive DSS treatment (Figure 4D‒F). Histological examination revealed less damage and reduced histological inflammation features in the Nsd2^Vil‐OE^ colons, in a sharp contrast to local inflammatory cell infiltration in the wild‐type colons (Figure 4G). Consistently, the colon of Nsd2^Vil‐OE^ mice also exhibited reduced immune response (Figure 4H) and contained significantly lower levels of proinflammatory cytokines compared to control mice (Figure 4I). Taken together, our results strongly demonstrated that NSD2 overexpression in IECs is highly protective against experimental IBD in vivo.

### 3.5 Loss of NSD2 exacerbates the inflammatory response in the intestine

**FIGURE 5:**
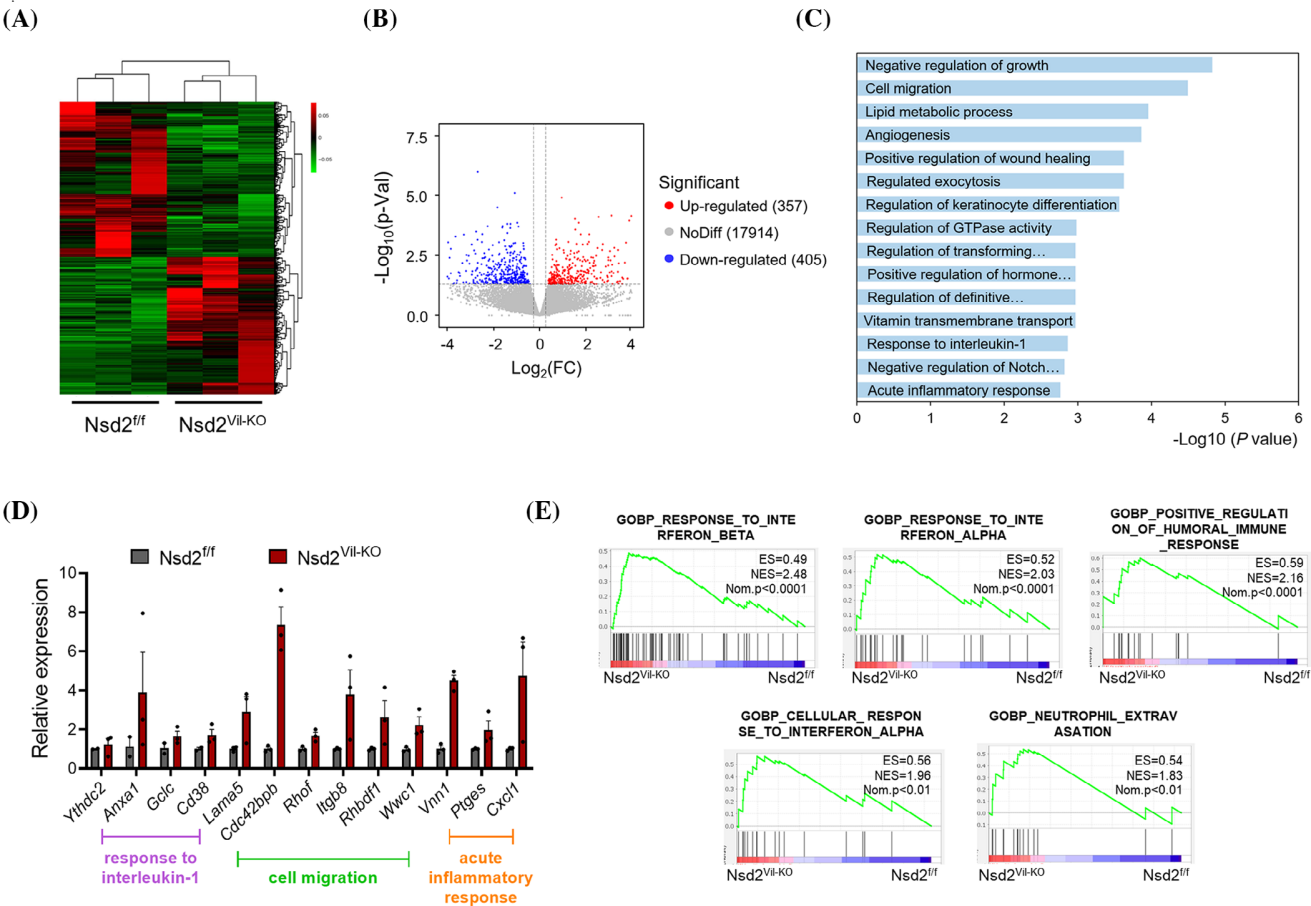
Loss of NSD2 exacerbates the inflammatory response in the intestine. (A) Heatmap of differentially expressed genes in intestinal epithelial cells (IECs) from 2% dextran sodium sulphate (DSS)‐treated Nsd2^f/f^ and Nsd2^Vil‐KO^ mice. (B) Volcano plot of comparative RNA sequencing (RNA‐seq) data between 2% DSS‐treated Nsd2^f/f^ and Nsd2^Vil‐KO^ mice. The *x*‐axis specifies the log_2_ fold changes (FC), and the *y*‐axis specifies the ‒log_10_ *p*‐value. Blue dots represent downregulated genes, and red dots represent upregulated genes. (C) Gene Ontology (GO) analysis of differentially expressed genes. (D) qRT‐PCR was used to verify the differentially expressed genes related to immune cell migration, response to interleukin‐1, and acute inflammatory response (*n* = 3 per group). (E) Gene set enrichment analysis (GSEA) enrichment plots of differentially expressed genes associated with Nsd2 deletion.

To gain an insight into the changes in biological processes and pathways caused by NSD2 loss, we subsequently performed RNA‐seq analysis using colon epithelial cells isolated from DSS‐treated Nsd2^f/f^ and Nsd2^Vil‐KO^ mice. The criterion for screening differentially expressed genes (DEGs) was a fold change (FC) >1.2, and a *p*‐value <.05 was considered indicative of significant differential gene expression. Compared to the Nsd2^f/f^ mice, RNA‐seq showed significant changes in the overall transcriptome of the Nsd2^Vil‐KO^ mice (Figure 5A). A total of 18 676 DEGs were identified, including 357 upregulated and 405 downregulated genes (Figure 5B). The Gene Ontology analysis for biological process‐associated genes revealed that DEGs were primarily enriched in immune cell migration, response to interleukin‐1, and acute inflammatory response, consistent with the earlier results of increased immune cell infiltration (Figure 5C). RT‐qPCR was performed and validated these findings (Figure 5D). We conducted gene set enrichment analysis, and the data indicated changes in inflammatory response, consistent with the RNA‐seq findings (Figure 5E). These data further confirmed that NSD2 loss exacerbates the inflammatory response in IECs.

### 3.6 NSD2 loss leads to a reduction in Fmo mRNA levels and impedes taurine accumulation

**FIGURE 6:**
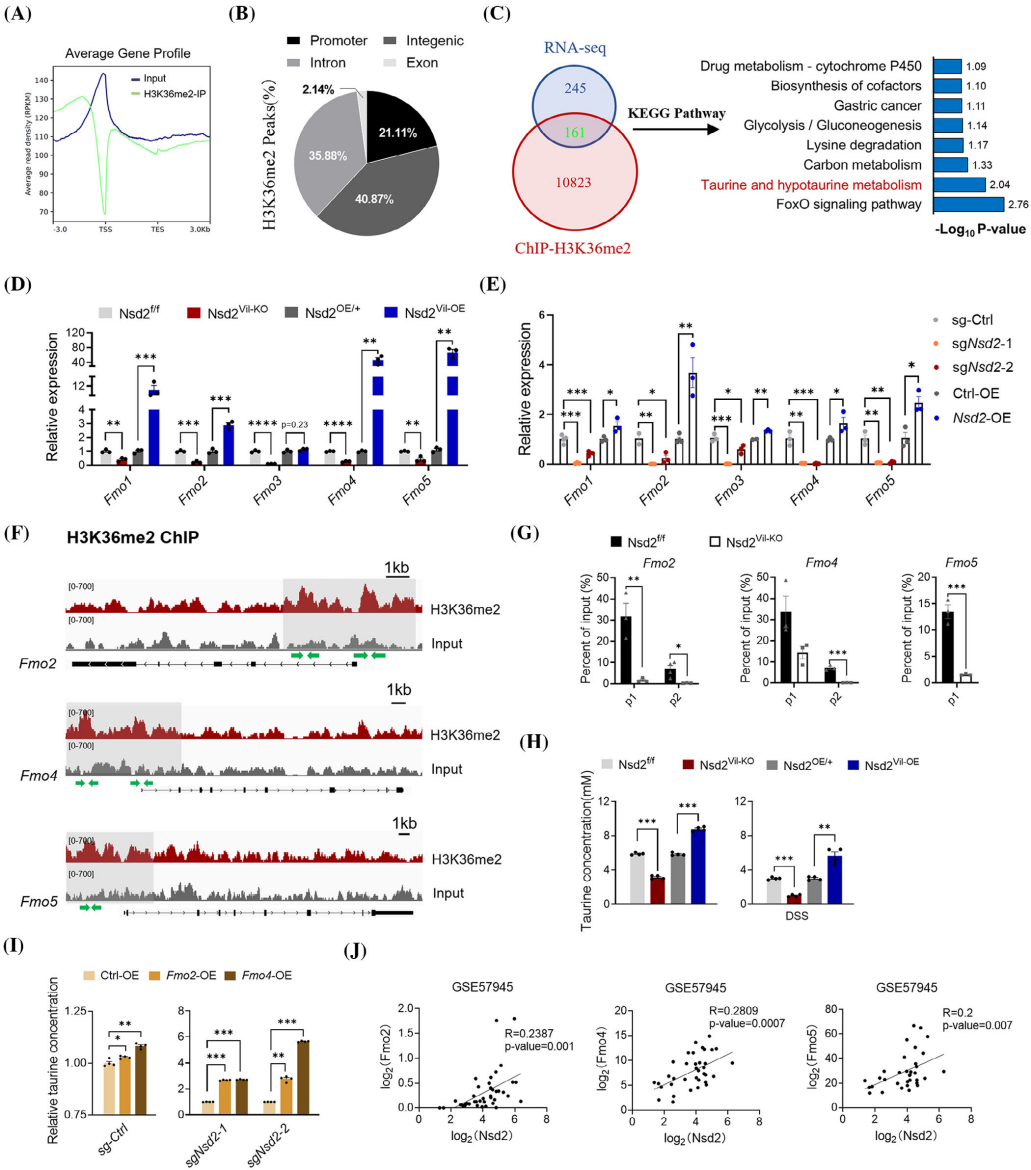
NSD2 loss leads to a reduction in Fmo mRNA levels and impedes taurine accumulation. (A) Normalised read density of H3K36me2 chromatin immunoprecipitation sequencing (ChIP‐seq) signals in colons from Nsd2^f/f^ and Nsd2^Vil‐KO^ mice, ranging from 3 kb upstream of the TSS to 3 kb downstream of the TES. (B) Mapping of H3K36me2 ChIP peaks relative to genomic annotations. (C) The overlap of significantly affected genes in ChIP‐seq and RNA sequencing (RNA‐seq) data, along with representative KEGG terms. (D) Quantitative mRNA expression levels of *Fmo1*, *Fmo2*, *Fmo3*, *Fmo4* and *Fmo5* in the colons of Nsd2^f/f^, Nsd2^Vil‐KO^, Nsd2^OE/+^ and Nsd2^Vil‐OE^ mice after dextran sodium sulphate (DSS) treatment (*n* = 3 for each group). (E) RT‐qPCR analysis of Fmo genes using sg‐Ctrl, sg*Nsd2*‐1, sg*Nsd2*‐2, Ctrl‐OE and *Nsd2*‐OE (SW620 derived) samples (*n* = 3 per group). Statistical analysis was performed using an unpaired *t*‐test. (F) Snapshot of H3K36me2 ChIP‐seq signals at the *Fmo2*, *Fmo4* and *Fmo5* gene loci in intestinal epithelial cells (IECs) from DSS‐treated (4 days) Nsd2^f/f^ and Nsd2^Vil‐KO^ mice. (G) ChIP‐qPCR of *Fmo2*, *Fmo4* and *Fmo5*. The location of the ChIP primer pairs used is denoted with a green arrow (*n* = 3 per group). (H) The level of taurine in IECs from Nsd2^Vil‐KO^ and Nsd2^Vil‐OE^ mice and their littermates, treated with or without DSS, was determined using a taurine microplate assay kit (*n* = 4). (I) The level of taurine in *Nsd2*‐deficient SW620 cell line transfected with *Fmo2* and *Fmo4* overexpression plasmids. (J) Correlation between *Nsd2* and *Fmo2*, *Fmo4* and *Fmo5* expression levels in inflammatory bowel disease (IBD) specimens (GSE 57945). Statistical significance was determined using the Pearson correlation coefficient. The data represent the mean ± SEM, and statistical significance was determined by a two‐tailed Student's *t*‐test unless otherwise indicated. ^*^ *p* < .05, ^**^ *p* < .01 and ^***^ *p* < .001; KEGG, Kyoto Encyclopedia of Genes and Genomes; N.S., not significant; SEM, standard error of the mean; TES. transcription end sites; TSS, transcription start sites.

To further explore the NSD2‐H3K36me2‐mediated mechanism, we performed ChIP sequencing (ChIP‐seq) targeting H3K36me2 in Nsd2^f/f^ and Nsd2^Vil‐KO^ IECs after 4 days of DSS treatment. The H3K36me2 ChIP‐seq analysis revealed a total of 10 984 filtered peaks, distributed across promoters (21.11%), introns (35.88%), intergenic regions (40.87%) and exons (2.14%; Figure 6A,B). To correlate the chromatin binding with the transcriptional regulation, the ChIP‐seq data were aligned with the expression profile. The Venn diagrams showed that 161 genes had expression changes after NSD2 ablation (Figure 6C). Kyoto Encyclopedia of Genes and Genomes (KEGG) analysis revealed that most of the DEGs are related to the FoxO signalling pathway and taurine and hypotaurine metabolism (Figure 6C). Further study showed no significant difference in the FoxO signalling pathway between Nsd2^Vil‐KO^ mice and control mice (Figure S3). Notably, the taurine and hypotaurine metabolism pathway was significantly enriched within our dataset. The data showed that *Fmo2*, *Fmo4* and *Fmo5* (flavin‐containing monooxygenase, which functions as the taurine‐synthesis enzyme) were downregulated in Nsd2^Vil‐KO^ mice. Furthermore, qPCR analysis demonstrated that the loss of NSD2 resulted in the downregulation of FMOs expression levels, while the overexpression of NSD2 led to their upregulation (Figure 6D). In addition, we conducted qPCR analysis in CRC cell lines SW620 and MC38 transfected with vector, sg*Nsd2*‐1, sg*Nsd2*‐2 and *Nsd2*‐OE plasmids. qPCR analysis revealed that NSD2 deletion significantly downregulated FMOs expression levels, while the expression levels of the FMOs were significantly restored by overexpressing NSD2 (Figures 6E and S4A). Additionally, ChIP‐seq tracks (Figure 6F) and ChIP‐qPCR (Figure 6G) confirmed that *Fmo2*, *Fmo4* and *Fmo5* exhibited decreased H3K36me2 occupancy specifically in their promoter regions, compared to controls.

Next, we found that the taurine concentration was decreased in sg*Nsd2* cells compared to the control mice, while the overexpression of NSD2 caused an increase in taurine concentration (Figure S4B). These data indicate a potential inhibition of taurine accumulation in the absence of NSD2. Furthermore, we also found a significant reduction in taurine concentration in NSD2‐depleted IECs with or without DSS treatment (Figure 6H). In contrast, a significant increase in taurine concentration was observed in Nsd2^Vil‐OE^ mice compared to controls (Figure 6H). To further examine whether the decreased taurine concentration caused by NSD2 deficiency could be rescued by Fmo overexpression, we transfected sg*Nsd2* SW620 cells with *Fmo2*‐OE and *Fmo4*‐OE plasmids. As anticipated, the overexpression of *Fmo2* and *Fmo4* elevated the taurine levels in both sg*Nsd2*‐1 and sg*Nsd2*‐2 cells (Figure 6I). Meanwhile, analysis of clinical IBD patient specimens indicated the positive correlations between the mRNA levels of *Nsd2* and *Fmo2*, *Fmo4* or *Fmo5*, respectively (Figure 6J). Taken together, these results collectively demonstrated that NSD2 loss leads to reductions in *Fmo* mRNA levels and impedes physiological taurine accumulation.

### 3.7 upplementation of taurine relieves intestinal epithelial damage in NSD2‐deficient IBD mouse

**FIGURE 7:**
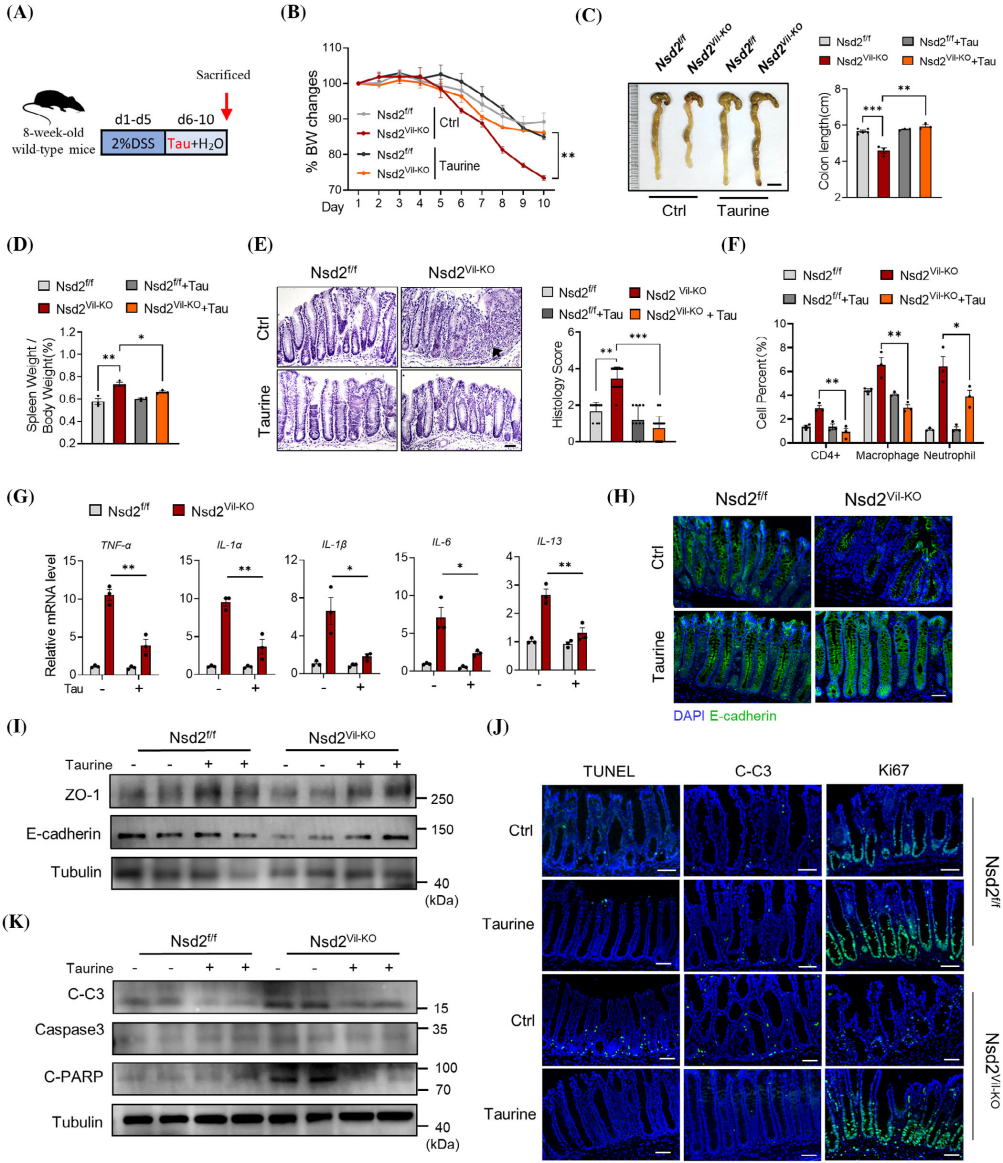
The supplementation of taurine relieves intestinal epithelial damage in NSD2‐deficient inflammatory bowel disease (IBD) mouse. (A) Schematic representation of the dextran sodium sulphate (DSS) treatment and taurine supplementation protocol. Relative percentage change of body weight (B), and representative images and quantification of colon morphology and lengths (C) of Nsd2^f/f^ and Nsd2^Vil‐KO^ mice after 2% DSS administration for 5 days, intragastric administration for 5 days, and sacrificed on day 10 (*n* = 3). (D) Spleen weight normalised to body weight at day 10. (E) Representative images of haematoxylin and eosin (H&E) and histology score (right) of distal colon of Nsd2^Vil‐KO^ mice with or without taurine supplementation. Scale bar: 50 µm. Arrow: immune cell infiltration. (F) After DSS treatment, colonic lamina propria cells were analysed by flow cytometry (*n* = 3). (G) Relative gene expression levels of *Tnf‐a*, *Il‐1a*, *Il‐1b*, *Il‐6* and *Il‐13* from colonic tissues of Nsd2^Vil‐KO^ mice with or without taurine supplementation. (H) Immunofluorescence of E‐cadherin staining of the distal colon. Scale bar: 50 µm. (I) Western blotting analysis of ZO‐1 and E‐cadherin of the distal colon. (J) TUNEL, C‐C3 and Ki67 staining of the distal colon from Nsd2^Vil‐KO^ mice with or without taurine supplementation and counts of the positive cells. Scale bar: 50 µm (*n* = 3 each group). (K) Western blotting analysis of C‐C3, caspase‐3 and C‐PARP; error bars show mean ± standard deviation (SD). *p*‐Values were determined by unpaired two‐tailed *t*‐test. For body weight curves, two‐way analysis of variance (ANOVA) analysis with Sidak's multiple comparisons test.

Given that NSD2 loss in IECs led to a lower FMO‐mediated taurine concentration, we next investigated whether supplementation of taurine could alleviate experimental colitis caused by NSD2 deficiency. We administered 2% DSS in the drinking water of Nsd2^f/f^ and Nsd2^Vil‐KO^ mice for 5 days and continued with 5‐day taurine supplementation by intragastric administration (Figure 7A). As expected, Nsd2^Vil‐KO^ mice showed a significant relief from IBD after supplementation of taurine, as indicated by body weight, colon length, the ratio of splenic weight to body weight and histological score (Figure 7B‒E). Furthermore, we observed a marked reduction in immune cell infiltration and a suppression of proinflammatory cytokine and chemokine induction in taurine‐supplemented Nsd2^Vil‐KO^ mice compared to that in control mice (Figure 7F,G). Additionally, taurine supplementation led to a recovered protein level of ZO‐1 and E‐cadherin in the distal colon tissues of Nsd2^Vil‐KO^ mice (Figure 7H,I), indicating a repair of the intestinal epithelial barrier.

Moreover, we found that the number of TUNEL‐ and C‐C3‐positive IECs was reduced in taurine‐treated Nsd2^Vil‐KO^ mice after DSS treatment (Figure 7J,K). Meanwhile, an increase in Ki67‐positive cells was observed in taurine‐treated Nsd2^Vil‐KO^ mice (Figure 7J), suggesting a restoration of epithelial self‐renewal capacity. Taken together, our results demonstrate that supplementation with taurine inhibits IEC apoptosis, enhances epithelial cell self‐renewal and attenuates the intestinal inflammation caused by NSD2 loss.

## 4 DISCUSSION

**FIGURE 8:**
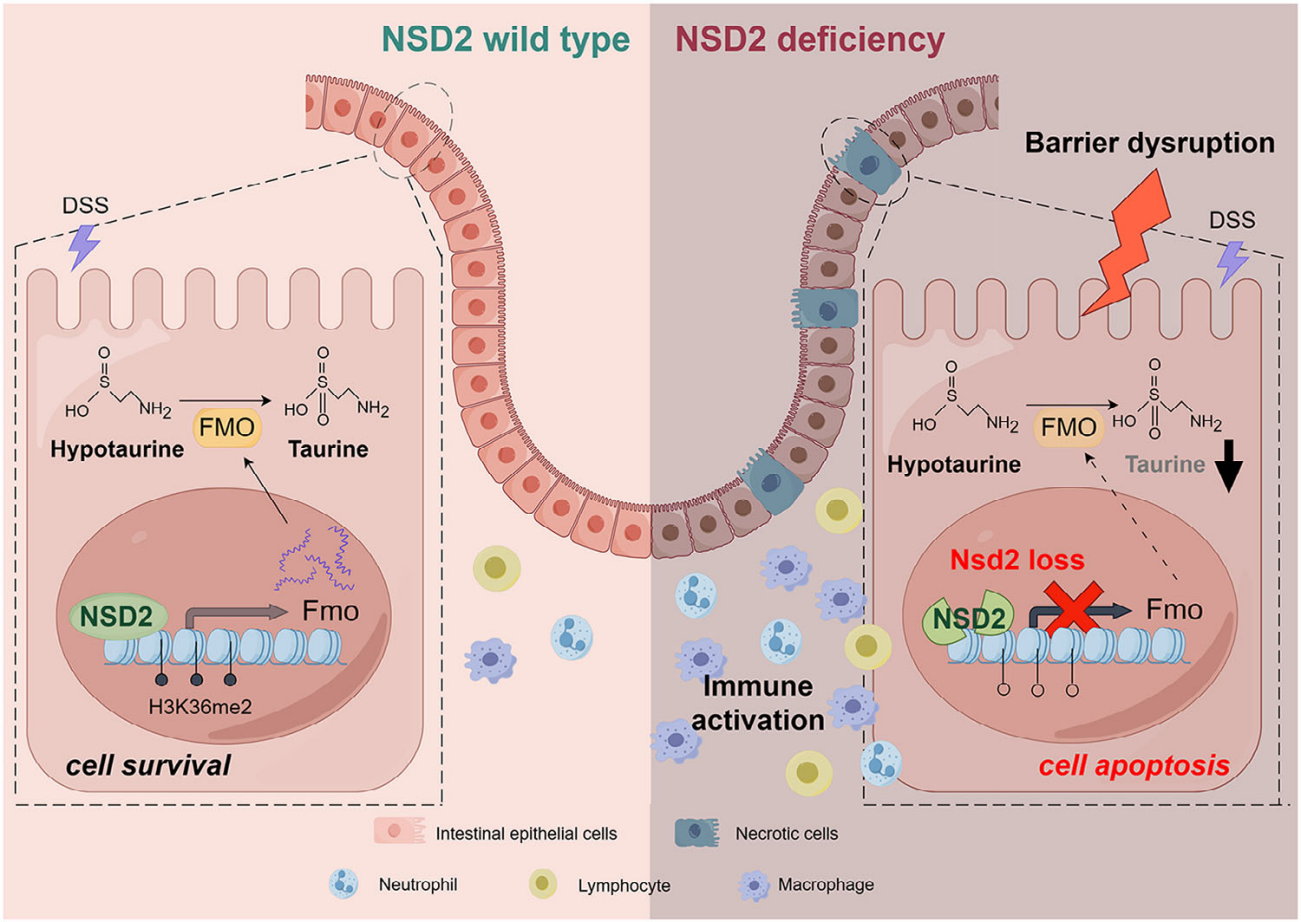
Schematic representation of the mechanism by which NSD2 drives flavin‐containing monooxygenase (FMO) in intestinal taurine accumulation to maintain intestinal homeostasis.

In this study, we reported that the deletion of NSD2 in IECs precipitates intestinal barrier dysfunction and exacerbates inflammatory infiltration. Our findings revealed a mechanistic link between NSD2 deficiency and downregulation of H3K36me2 at protein level, along with diminished Fmo expression at mRNA level, leading to impeded taurine accumulation in both in vitro and in vivo models. Supplementation of taurine can effectively alleviate intestinal epithelial damage in NSD2‐deficient IBD mice. Thus, these results indicate that NSD2 act as a protector of the intestinal epithelial barrier during intestinal inflammation, which helps maintain intestinal epithelial homeostasis by preventing cell apoptosis (Figure 8).

NSD2, a member of nuclear receptor‐binding SET domain protein (NSD) family, plays a crucial role in gene transcription, DNA replication and cellular differentiation. Several studies have shown the significant importance of NSD2 in chromatin regulation and regulates cell senescence, proliferation, migration, invasion and epithelial‒mesenchymal transition.^15^, ^28^, ^29^, ^30^, ^31^, ^32^, ^33^, ^34^, ^35^, ^36^, ^37^, ^38^, ^39^, ^40^, ^41^ Previous studies suggest that NSD2‐mediated H3K36me2 is crucial for transcription activation and the expression of multiple oncogenes in CRC.^30^, ^35^, ^42^, ^43^ These studies imply that NSD2, as a histone modifier, may play different roles depending on the genetic environment or context. Till now, there is limited documentation about the role of NSD2 in IBD. Our study highlights the protective role of NSD2 in IECs, where it safeguards against excessive apoptosis and facilitate the maintenance of intestinal epithelial homeostasis through FMO‐mediated physiological taurine metabolism.

Taurine, a conditionally essential beta‐amino, is an essential nutrient for the development, growth and maintenance of physiological functions of various tissues.^44^ Low taurine concentration is closely correlated with several age‐related diseases^45^ and taurine deficiency causes functional impairments in skeletal muscle, eye and the central nervous system.^46^ Moreover, taurine supplementation could enhance resistance to infection, prevent diabetes mellitus, insulin resistance and its complications, ischaemic stroke and inflammation.^18^, ^47^, ^48^, ^49^ The latest studies reported that taurine metabolism influences the proliferative, anti‐apoptotic and migratory abilities of cancer cells.^19^ Taurine can be obtained from the diet and absorbed by cells through taurine transporters.^50^ Due to limited exogenous intake, the maintenance of abundant taurine content in the mammalian body also originates from endogenous synthesis.^51^ Previous studies have shown that endogenous taurine is primarily produced from cysteine through the action of cysteine sulphonic acid decarboxylase.^52^ In addition to the cysteic acid pathway, hypotaurine can be oxygenated to taurine by FMOs,^51^ which is currently considered the primary pathway for taurine synthesis.^51^ Therefore, FMOs are essential for maintaining endogenous taurine synthesis. FMOs, which include subtypes 1–5, function as the taurine‐synthesis enzyme that converts the precursor hypotaurine to taurine.^51^, ^53^, ^54^ Additionally, FMOs are promiscuous enzymes that metabolise a wide range of exogenous compounds.^55^ There were significant differences in the expression levels of FMOs based on tissue, age, sex and species. In humans and mice, FMOs are primarily expressed in the liver and skin, and to a lesser extent in other tissues, such as the small intestine.^53^ Notably, it is reported that FMO5 play important roles in intestinal inflammation.^18^, ^56^, ^57^ Emerging research offers novel insights indicating that the FMO family is subject to modulation by a range of factors influencing both transcriptional and post‐transcriptional mechanisms. These factors encompass the precursor amino acids of taurine, hormonal influences, bile acids and cytokines.^51^ However, little is known about the epigenetic mechanism of FMOs regulation. Here, we found that the histone methyltransferase NSD2 can catalyse the H3K36me2 on the promoters of *Fmo2*, *Fmo4* and *Fmo5* (flavin‐containing monooxygenase, which functions as the taurine‐synthesis enzyme), thereby elevating the transcriptional levels of FMOs, leading to increased taurine synthesis and, consequently, a reinforced intestinal barrier during inflammation. Furthermore, overexpression of NSD2 in IECs significantly elevated FMOs levels and taurine concentration, effectively mitigating DSS‐induced IBD. In vitro experiments also confirmed these results. Taurine supplementation inhibited IEC apoptosis and assisted in mucosal repair, thereby effectively attenuating the intestinal inflammation caused by NSD2 loss.

Previous studies have shown that histone modification modulates the intestinal immune microenvironment.^58^, ^59^, ^60^, ^61^ Our study contributes to this field by pointing out the role of NSD2‐mediated H3K36me2 in IBD pathogenesis. Here, we found that NSD2‐mediated H3K36me2 protects IECs from excessive apoptosis by modulating FMOs transcriptional levels and preserving taurine concentration, thus maintaining an intact and functional mucosal barrier. It is also worthy to point out that NSD2 catalyses H3K36me2 at intergenic regions,^62^ which is crucial for the recruitment of DNMT3A and the maintenance of DNA methylation at these regions. However, the potential impact of NSD2 loss on DNA methylation patterns in intergenic regions, and its subsequent effects on Fmo gene expression, needs to be further studied with whole‐genome bisulphite sequencing in the future.

In summary, our study delineates a novel role for NSD2 in the intestinal inflammatory diseases. We propose that NSD2's epigenetic regulation of FMOs and its impact on taurine metabolism are critical for intestinal epithelial barrier function and homeostasis. These findings provide therapeutic insights into potential therapeutic approaches for IBD and possibly other human disorders associated with NSD2 deficiency.

## AUTHOR CONTRIBUTIONS

Li Li and Wei‐Qiang Gao conceived the experimental concept, designed the experiments and interpreted the data. Yue Xu performed most of the experiments and wrote the manuscript. Xiuying Xiao performed tissue microarray and pathology analyses. Chunxiao Ma, Ziyi Wang, Wenxin Feng, Wei Zhang, Ningyuan Liu and Rebiguli Aji assisted in some experiments. Xiangjun Meng helped with valuable discussion. Wei‐Qiang Gao assisted in some discussion. Li Li provided the overall guidance. All authors read and approved the final manuscript.

## CONFLICT OF INTEREST STATEMENT

The authors declare they have no conflicts of interest.

## ETHICS STATEMENT

Not applicable.

## Supporting information



Supporting Information

Supporting Information

## Data Availability

The authors declare that all data supporting the findings in this study are available within the paper, Supporting Information and source data. All data are available from the authors upon reasonable request. RNA‐seq and ChIP‐seq raw data have been deposited in the Gene Expression Omnibus under accession numbers: GSE267227 and GSE267102.
