# Supplementary material for: Epithelial NSD2 maintains FMO‐mediated taurine biosynthesis to prevent intestinal barrier disruption
Source: Clin Transl Med. 2024 Dec 10;14(12):e70128. doi: 10.1002/ctm2.70128 (PMC11835373; doi:10.1002/ctm2.70128)

Figure S1 NSD2 deletion does not affect the self-renew and differentiation of IECs under steady state.

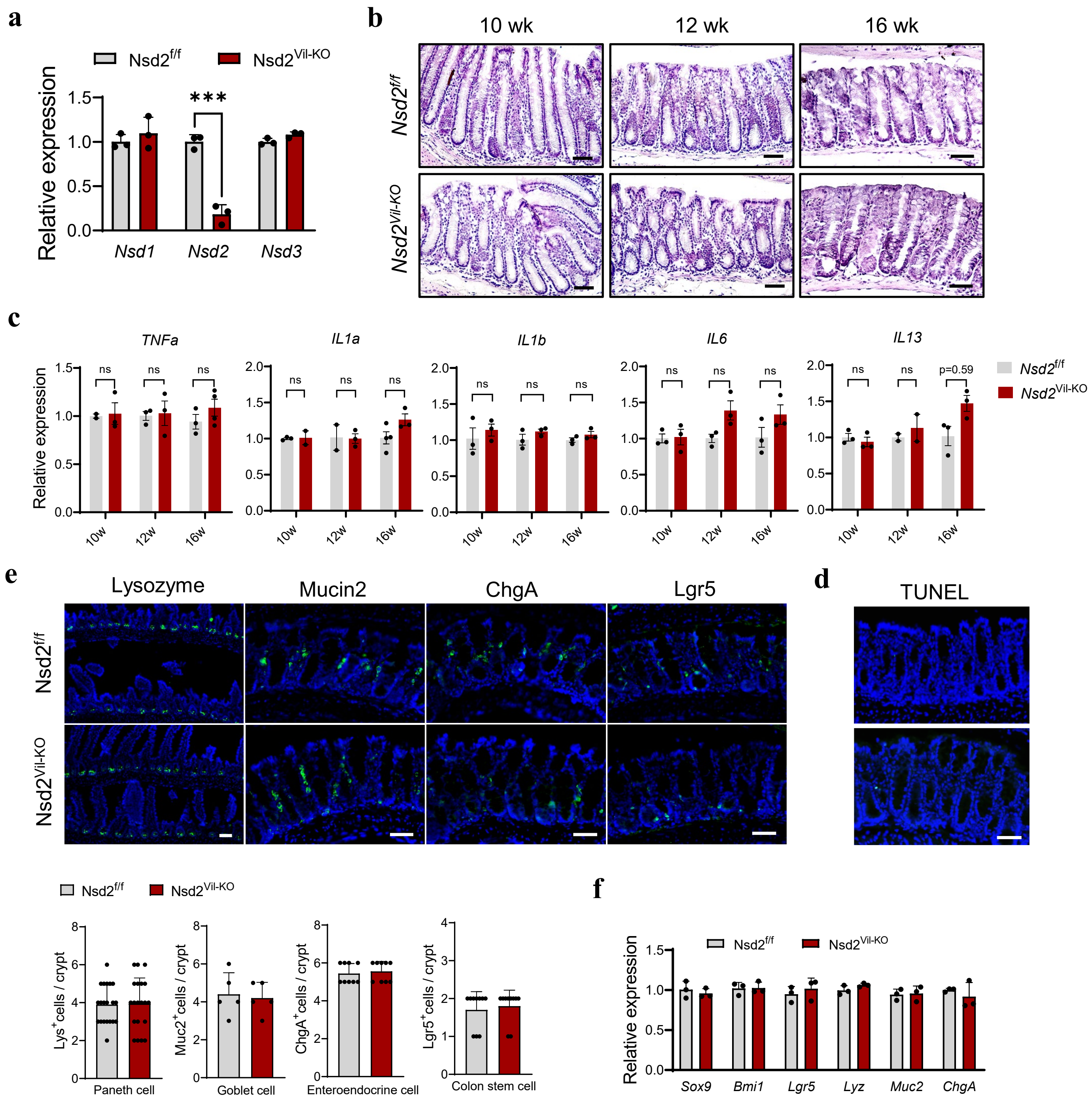

Figure S2 NSD2 deletion results in a loss of IECs after DSS treatment.

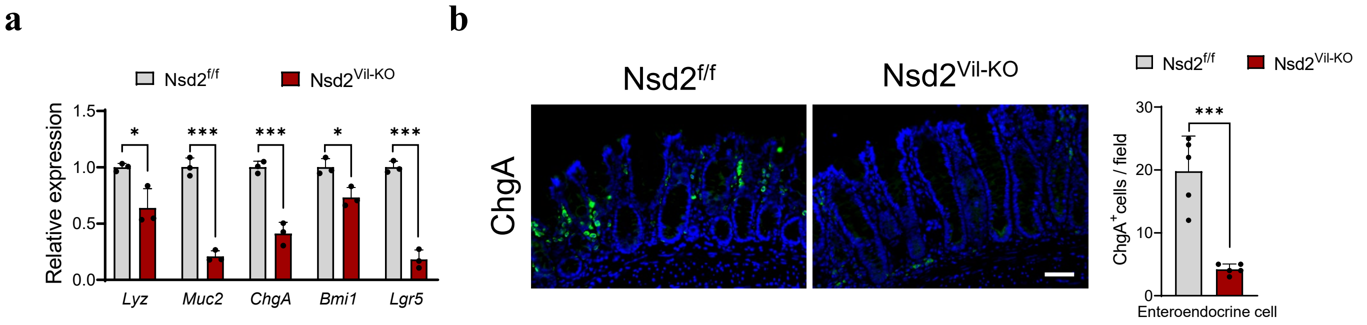

Figure S3 Adult NSD2 loss does not affect FoxO pathway in the colons.

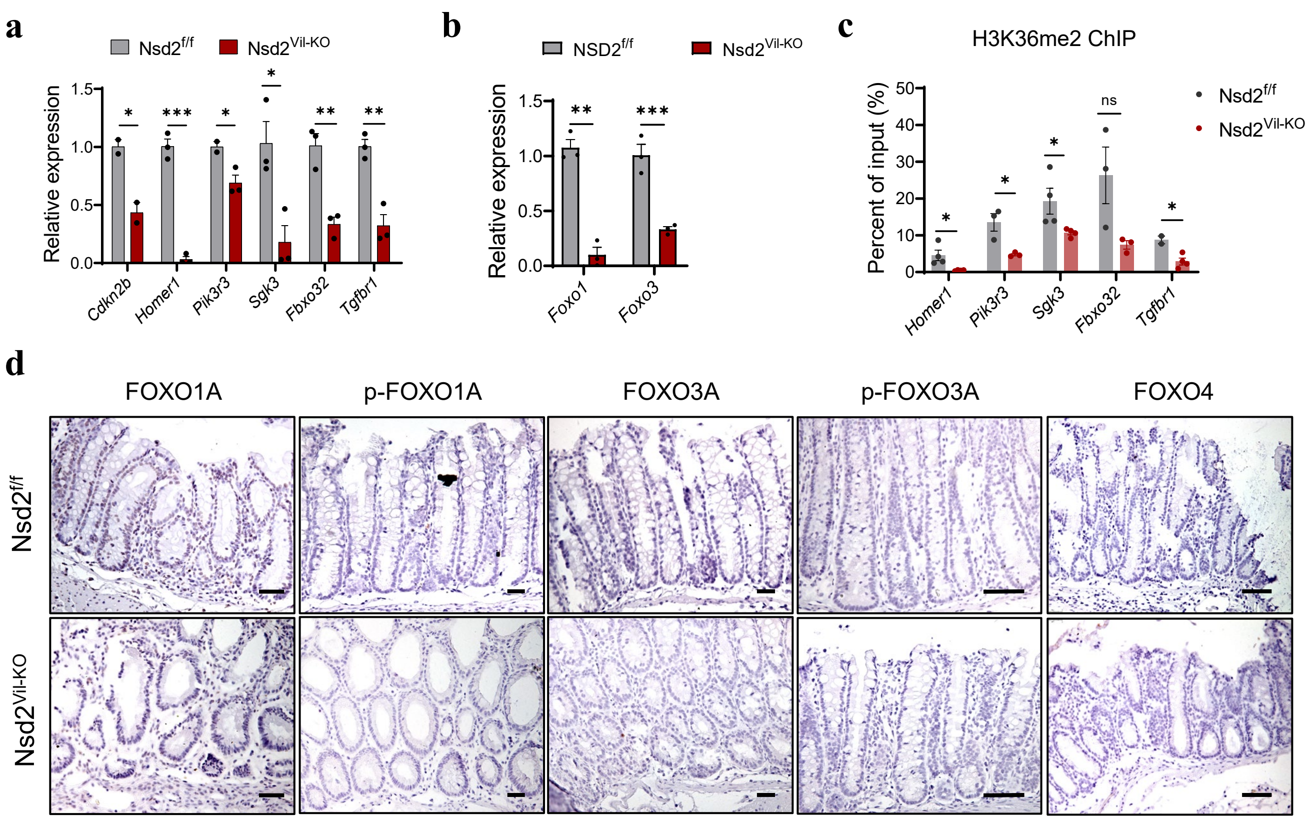

Figure S4 Loss of NSD2 reduces Fmo RNA levels and impedes taurine accumulation in vitro.

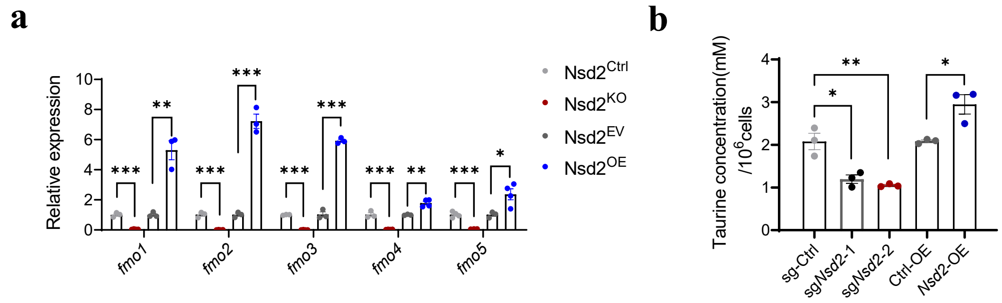

Supplement: Supplementary file 1 — Supporting Information [file CTM2-14-e70128-s001.pdf]
